# Supplementary material for: Remodelling of cystic fibrosis respiratory microbiota in response to extended elexacaftor–tezacaftor–ivacaftor therapy
Source: Microbiome. 2026 May 30;14:192. doi: 10.1186/s40168-026-02440-7 (PMC13430856; doi:10.1186/s40168-026-02440-7)
Supplement: Supplementary file 2 — Supplementary Material 1: Figure S1 Comparisons of microbiota characteristics between cough swab and sputum samples. (A) number of sequence reads between sample types, (B, C, D) comparisons of diversity and dominance, and (E) comparisons of within and between sample type microbiota compositional similarity. Boxplots show 25-75th interquartile (IQR) range with whiskers showing 1.5 times IQR. Purple crosses represent the mean in each group. Circles denote individual measures within a given group. Swab n = 102 and sputum n = 352. Mean and standard deviation of the mean values: (A) swab = 21632.3 ± 17893.8 and sputum 70333.7 ± 99340.8; (B) swab 8.04 ± 5.81, sputum 9.76 ± 8.60; (C) swab 1.44 ± 0.73, sputum 1.41 ± 0.90; (D) swab 0.44 ± 0.24, sputum 0.48 ± 0.27; and (E) swab 0.26 ± 0.15, sputum 0.26± 0.16, between group 0.22 ± 0.14 (Pairwise comparisons: swab = 5151, sputum = 61776, between groups = 35904). Kruskal-Wallis test statistics (H) and significance (P): (A) H = 51.81, P <0.0001; (B) H = 0.341, P =0.559; (C) H = 0.133, P = 0.715; (D) H = 1.115, P =0.291; and (E) H = 0.271, P = 0.603. [file 40168_2026_2440_MOESM1_ESM.docx]

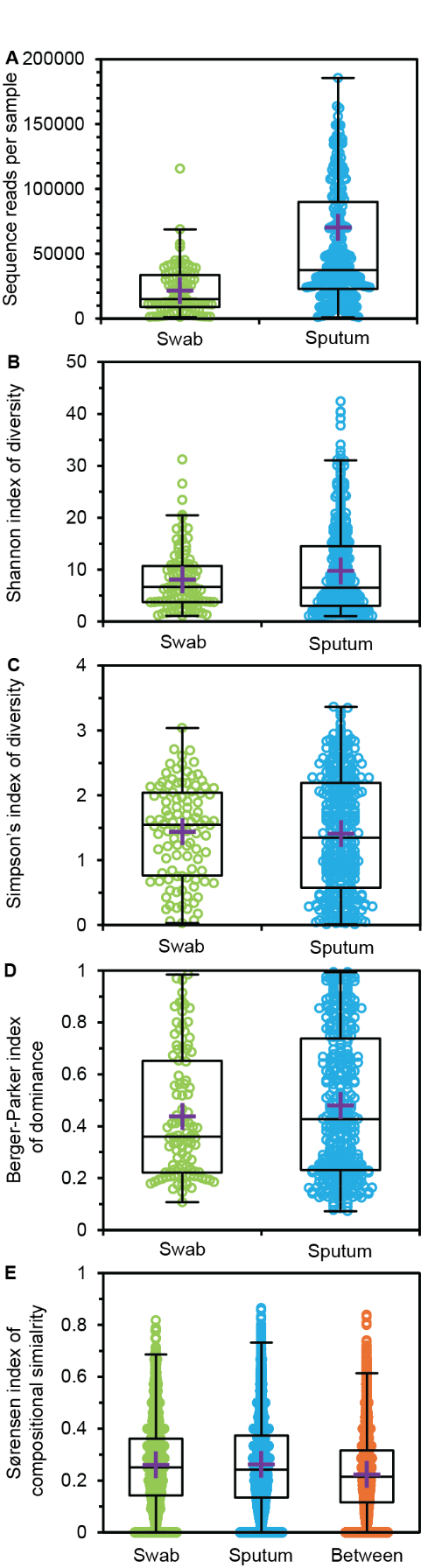


**Supplementary Figure 1** Comparisons of microbiota characteristics between cough swab and sputum samples. (A) number of sequence reads between sample types, (B, C, D) comparisons of diversity and dominance, and (E) comparisons of within and between sample type microbiota compositional similarity. Boxplots show 25-75^th^ interquartile (IQR) range with whiskers showing 1.5 times IQR. Purple crosses represent the mean in each group. Circles denote individual measures within a given group. Swab *n* = 102 and sputum *n* = 352. Mean and standard deviation of the mean values: (A) swab = 21632.3 ± 17893.8 and sputum 70333.7 ± 99340.8; (B) swab 8.04 ± 5.81, sputum 9.76 ± 8.60; (C) swab 1.44 ± 0.73, sputum 1.41 ± 0.90; (D) swab 0.44 ± 0.24, sputum 0.48 ± 0.27; and (E) swab 0.26 ± 0.15, sputum 0.26 ± 0.16, between group 0.22 ± 0.14 (Pairwise comparisons: swab = 5151, sputum = 61776, between groups = 35904). Kruskal-Wallis test statistics (*H*) and significance (*P*): (A) *H* = 51.81, *P* <0.0001; (B) *H* = 0.341, *P* =0.559; (C) *H* = 0.133, *P* = 0.715; (D) *H* = 1.115, *P* =0.291; and (E) *H* = 0.271, *P* = 0.603.
