# Supplementary material for: Remodelling of cystic fibrosis respiratory microbiota in response to extended elexacaftor–tezacaftor–ivacaftor therapy
Source: Microbiome. 2026 May 30;14:192. doi: 10.1186/s40168-026-02440-7 (PMC13430856; doi:10.1186/s40168-026-02440-7)
Supplement: Supplementary file 3 — Supplementary Material 2: Figure S2 Comparison of microbiota compositional similarities between pre- and on-elexacaftor/tezacaftor/ivacaftor (ETI) sample groups. Respiratory microbiota samples from adults with cystic fibrosis (CF) at (A) 6 months, (B) 1 year, (C) 2 years, and (D) 3 years of therapy duration compared to pre-ETI samples stratified by disease severity, along with samples from a non-CF healthy cohort. Compositional similarities were measured using the Sørensen index of similarity. Boxplots show 25-75th interquartile (IQR) range with whiskers showing 1.5 times IQR. Purple crosses represent the mean in each group. In each instance, circles denote compositional similarities of samples taken pairwise between each given pairing of groups. Summary statistics for PERMANOVA tests between all groups taken pairwise are provided in Supplementary Table S3. [file 40168_2026_2440_MOESM2_ESM.docx]

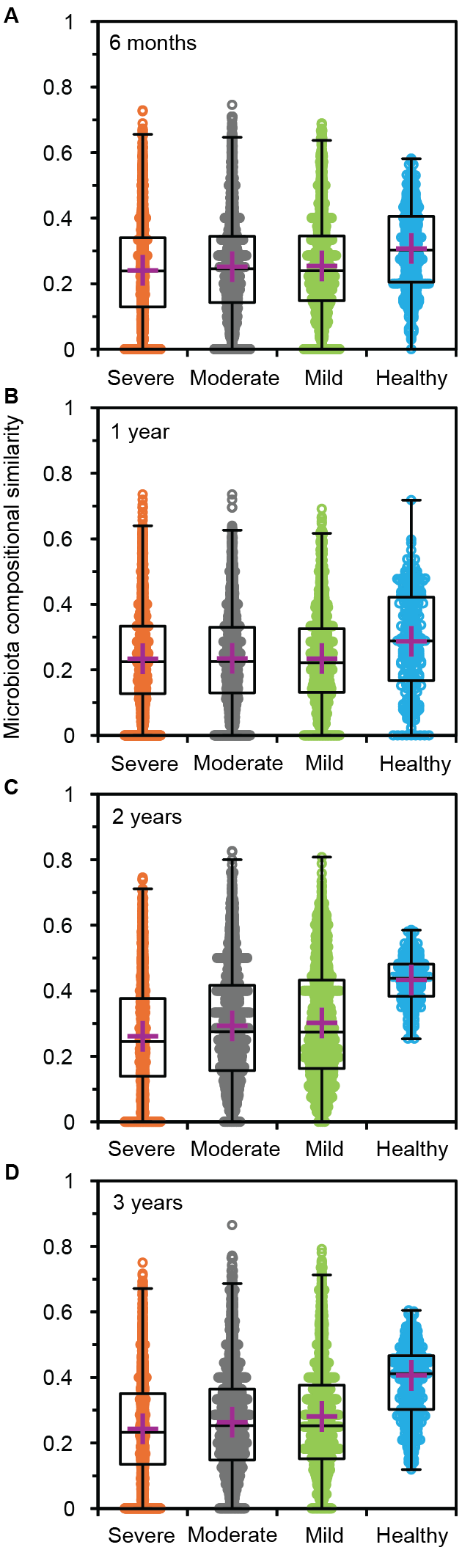


**Figure S2** Comparison of microbiota compositional similarities between pre- and on-elexacaftor/tezacaftor/ivacaftor (ETI) sample groups. Respiratory microbiota samples from adults with cystic fibrosis (CF) at (A) 6 months, (B) 1 year, (C) 2 years, and (D) 3 years of therapy duration compared to pre-ETI samples stratified by disease severity, along with samples from a non-CF healthy cohort. Compositional similarities were measured using the Sørensen index of similarity. Boxplots show 25-75^th^ interquartile (IQR) range with whiskers showing 1.5 times IQR. Purple crosses represent the mean in each group. In each instance, circles denote compositional similarities of samples taken pairwise between each given pairing of groups. Summary statistics for PERMANOVA tests between all groups taken pairwise are provided in Supplementary Table S3.
