# Supplementary material for: Remodelling of cystic fibrosis respiratory microbiota in response to extended elexacaftor–tezacaftor–ivacaftor therapy
Source: Microbiome. 2026 May 30;14:192. doi: 10.1186/s40168-026-02440-7 (PMC13430856; doi:10.1186/s40168-026-02440-7)
Supplement: Supplementary file 4 — Supplementary Material 3: Figure S3 Distribution and abundance of bacterial taxa across adults with CF receiving Azithromycin treatment or not when either pre-ETI or on-ETI therapy. (A) Pre-ETI therapy all samples, (B) On-ETI therapy all samples, (C) and (D) pre-ETI and on-ETI without Azithromycin, and (E) and (F) pre-ETI and on-ETI with Azithromycin. Given is the percentage number of patient respiratory samples each bacterial taxon was observed to be distributed across, plotted against the mean percentage abundance across those samples. Core taxa are defined as those are in >50% of samples (orange circles), and satellite taxa (grey circles) defined as those that do not. Canonical CF pathogens are highlighted in each plot. Distribution-abundance relationship regression statistics: (A) R2 = 0.62, F1,430 = 268.4, P < 0.0001; (B) R2 = 0.70, F1,377= 494.7, P < 0.0001; (C) R2 = 0.39, F1,273 = 171.5, P < 0.0001; (D) R2 = 0.53, F1,258 = 293.4, P < 0.0001; (E) R2 = 0.44, F1,374 = 288.3, P < 0.0001; and (F) R2 = 0.61, F1,324 = 502.2, P < 0.0001. Core taxa are listed in Supplementary Table S5.. [file 40168_2026_2440_MOESM3_ESM.docx]

**
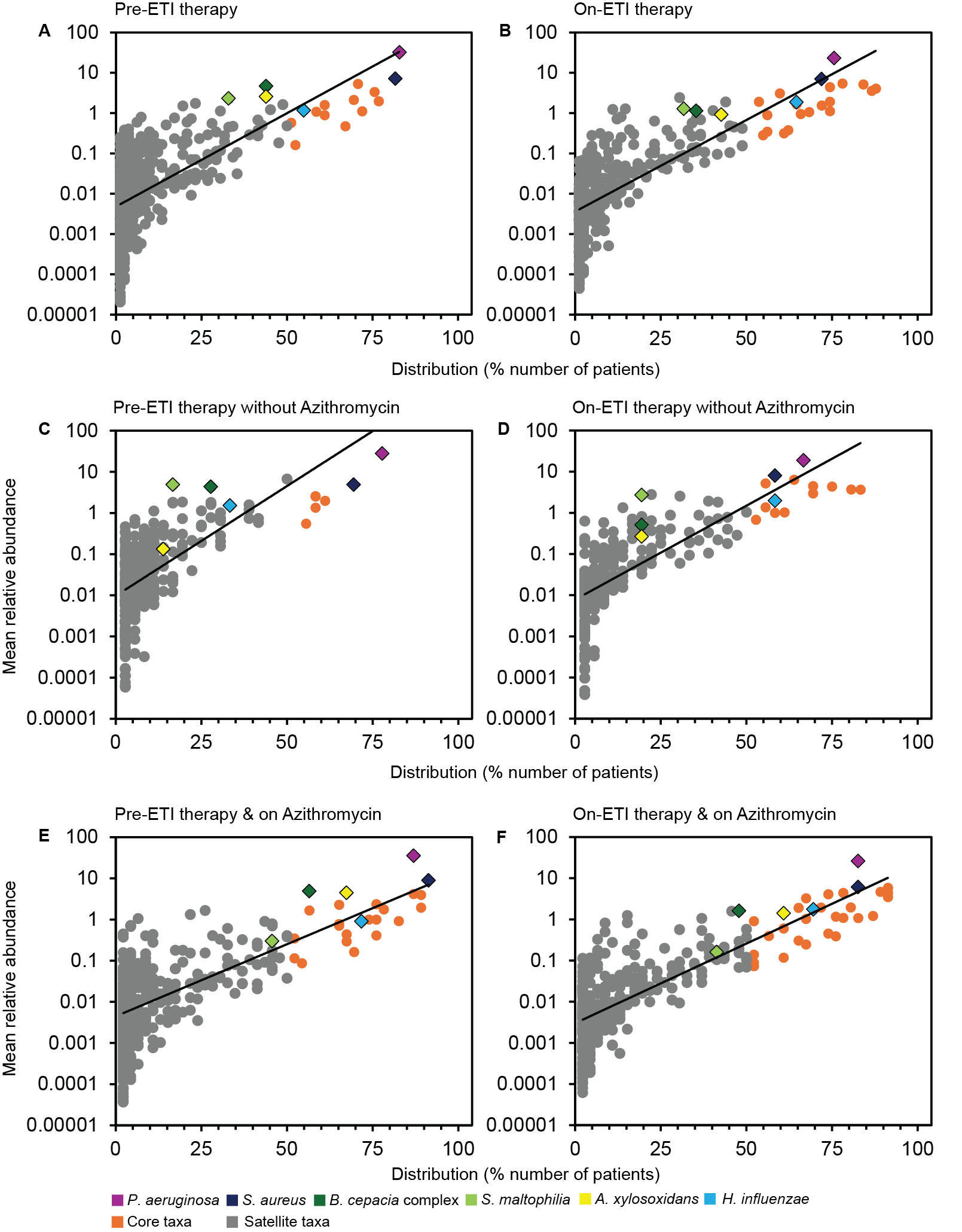
**

**Figure S3** Distribution and abundance of bacterial taxa across adults with CF receiving Azithromycin treatment or not when either pre-ETI or on-ETI therapy. (**A**) Pre-ETI therapy all samples, (**B**) On-ETI therapy all samples, (**C**) and (**D**) pre-ETI and on-ETI without Azithromycin, and (**E**) and (**F**) pre-ETI and on-ETI with Azithromycin. Given is the percentage number of patient respiratory samples each bacterial taxon was observed to be distributed across, plotted against the mean percentage abundance across those samples. Core taxa are defined as those are in >50% of samples (orange circles), and satellite taxa (grey circles) defined as those that do not. Canonical CF pathogens are highlighted in each plot. Distribution-abundance relationship regression statistics: (**A**) *R*^2^ = 0.62, *F*_1,430_ = 268.4, *P* < 0.0001; (**B**) *R*^2^ = 0.70, *F*_1,377_ = 494.7, *P* < 0.0001; (**C**) *R*^2^ = 0.39, *F*_1,273_ = 171.5, *P* < 0.0001; (**D**) *R*^2^ = 0.53, *F*_1,258_ = 293.4, *P* < 0.0001; (**E**) *R*^2^ = 0.44, *F*_1,374_ = 288.3, *P* < 0.0001; and (**F**) *R*^2^ = 0.61, *F*_1,324_ = 502.2, *P* < 0.0001. Core taxa are listed in Supplementary Table S5.
