# Supplementary material for: Remodelling of cystic fibrosis respiratory microbiota in response to extended elexacaftor–tezacaftor–ivacaftor therapy
Source: Microbiome. 2026 May 30;14:192. doi: 10.1186/s40168-026-02440-7 (PMC13430856; doi:10.1186/s40168-026-02440-7)
Supplement: Supplementary file 5 — Supplementary Material 4: Table S1 Kruskal-Wallis summary statistics for all groups taken pairwise. Summary statistics are given for (A) Shannon index of diversity, (B) Simpson’s index of diversity, and (C) Berger-Parker index of dominance (d). For each index, test statistic H observed is given in the upper triangle and significance (P) in the lower triangle. H critical was 0.384. Tests with significant differences are highlighted in green. [file 40168_2026_2440_MOESM4_ESM.docx]

**Table S1** Kruskal-Wallis summary statistics for all groups taken pairwise. Summary statistics are given for (A) Shannon index of diversity, (B) Simpson’s index of diversity, and (C) Berger-Parker index of dominance (*d*). For each index, test statistic *H* observed is given in the upper triangle and significance (*P*) in the lower triangle. Tests with significant differences are highlighted in green.

| **A** |  | **Pre-ETI** | |  | **Non-CF** | **On-ETI** |  |  |  |
| --- | --- | --- | --- | --- | --- | --- | --- | --- | --- |
|  |  | **Severe** | **Moderate** | **Mild** | **Healthy** | **6M** | **1Y** | **2Y** | **3Y** |
| **Pre-ETI** | **Severe** |  | 0.072 | 3.991 | 18.904 | 0.796 | 1.461 | 22.629 | 9.928 |
|  | **Moderate** | 0.789 |  | 4.307 | 19.292 | 0.883 | 2.690 | 26.518 | 12.553 |
|  | **Mild** | 0.045 | 0.038 |  | 17.128 | 0.067 | 0.001 | 10.862 | 5.328 |
| **Non-CF** | **Healthy** | <0.0001 | <0.0001 | <0.0001 |  | 13.994 | 13.092 | 7.400 | 7.552 |
| **On-ETI** | **6M** | 0.372 | 0.267 | 0.795 | <0.0001 |  | 0.229 | 7.775 | 4.629 |
|  | **1Y** | 0.227 | 0.101 | 0.973 | <0.0001 | 0.585 |  | 7.890 | 3.912 |
|  | **2Y** | <0.0001 | <0.0001 | <0.0001 | 0.007 | 0.005 | 0.005 |  | 0.452 |
|  | **3Y** | 0.002 | <0.0001 | 0.021 | 0.006 | 0.031 | 0.046 | 0.501 |  |
|  |  |  |  |  |  |  |  |  |  |
| **B** |  |  |  |  |  |  |  |  |  |
| **Simpson's index** | | **Pre-ETI** | |  | **Non-CF** | **On-ETI** |  |  |  |
|  |  | **Severe** | **Moderate** | **Mild** | **Healthy** | **6M** | **1Y** | **2Y** | **3Y** |
| **Pre-ETI** | **Severe** |  | 0.096 | 3.968 | 15.597 | 0.379 | 1.535 | 17.975 | 9.193 |
|  | **Moderate** | 0.756 |  | 5.024 | 16.824 | 0.883 | 2.593 | 21.768 | 11.252 |
|  | **Mild** | 0.047 | 0.025 |  | 13.298 | 0.407 | 0.001 | 14.733 | 4.353 |
| **Non-CF** | **Healthy** | <0.0001 | <0.0001 | <0.0001 |  | 12.522 | 10.982 | 3.717 | 4.343 |
| **On-ETI** | **6M** | 0.539 | 0.347 | 0.524 | <0.0001 |  | 0.536 | 11.67 | 5.465 |
|  | **1Y** | 0.215 | 0.107 | 0.992 | <0.0001 | 0.464 |  | 9.249 | 3.923 |
|  | **2Y** | <0.0001 | <0.0001 | 0.001 | 0.054 | 0.001 | 0.002 |  | 1.073 |
|  | **3Y** | 0.002 | 0.001 | 0.037 | 0.037 | 0.002 | 0.048 | 0.300 |  |
|  |  |  |  |  |  |  |  |  |  |
| **C** |  |  |  |  |  |  |  |  |  |
| **Berger-Parker** | | **Pre-ETI** | |  | **Non-CF** | **On-ETI** |  |  |  |
|  |  | **Severe** | **Moderate** | **Mild** | **Healthy** | **6M** | **1Y** | **2Y** | **3Y** |
| **Pre-ETI** | **Severe** |  | 0.171 | 2.526 | 12.667 | 0.348 | 1.205 | 21.244 | 8.700 |
|  | **Moderate** | 3.841 |  | 5.995 | 14.183 | 1.286 | 2.806 | 27.959 | 11.615 |
|  | **Mild** | 0.112 | 0.014 |  | 10.214 | 0.384 | 0.023 | 17.165 | 4.135 |
| **Non-CF** | **Healthy** | <0.0001 | <0.0001 | <0.0001 |  | 8.836 | 9.056 | 0.628 | 2.795 |
| **On-ETI** | **6M** | 0.555 | 0.257 | 0.535 | 0.003 |  | 0.329 | 12.379 | 4.990 |
|  | **1Y** | 0.272 | 0.094 | 0.879 | 0.003 | 0.566 |  | 11.601 | 3.901 |
|  | **2Y** | <0.0001 | <0.0001 | <0.0001 | 0.428 | <0.0001 | 0.001 |  | 1.868 |
|  | **3Y** | 0.003 | 0.001 | 0.042 | 0.095 | 0.025 | 0.048 | 0.172 |  |
