# Supplementary material for: Remodelling of cystic fibrosis respiratory microbiota in response to extended elexacaftor–tezacaftor–ivacaftor therapy
Source: Microbiome. 2026 May 30;14:192. doi: 10.1186/s40168-026-02440-7 (PMC13430856; doi:10.1186/s40168-026-02440-7)
Supplement: Supplementary file 6 — Supplementary Material 5: Table S2 Core taxa within each on-ETI therapy duration category. (A) 6 months duration, (B) 1 year, (C) 2 years, and (D) 3 years duration on ETI therapy. Given is distribution, the percentage number of samples a given core taxon was detected in, and average relative abundance across those samples. Given the length of the ribosomal sequences analysed, species identities should be considered putative. In each instance, taxa are ordered from most to least persistent. Canonical CF pathogens are highlighted in bold. [file 40168_2026_2440_MOESM5_ESM.docx]

**Table S2** Core taxa within each on-ETI therapy duration category**.** (**A**) 6 months duration, (**B**) 1 year, (**C**) 2 years, and (**D**) 3 years duration on ETI therapy. Given is distribution, the percentage number of samples a given core taxon was detected in, and average relative abundance across those samples. Given the length of the ribosomal sequences analysed, species identities should be considered putative. In each instance, taxa are ordered from most to least persistent. Canonical CF pathogens are highlighted in bold.

| **(A)** 6 months |  |  | **(B)** 1 year |  |  | **(C)** 2 years |  |  | **(D)** 3 years |  |  |
| --- | --- | --- | --- | --- | --- | --- | --- | --- | --- | --- | --- |
| **Taxon** | **Distribution** | **Abundance** | **Taxon** | **Distribution** | **Abundance** | **Taxon** | **Distribution** | **Abundance** | **Taxon** | **Distribution** | **Abundance** |
| *Rothia mucilaginosa* | 93.5 | 3.7 | *Veillonella dispar* | 89.7 | 6.3 | *Streptococcus parasanguinis* | 87.5 | 3.2 | *Streptococcus parasanguinis* | 100.0 | 7.5 |
| ***Pseudomonas aeruginosa*** | 87.0 | 30.5 | *Streptococcus salivarius* | 87.2 | 11.7 | *Streptococcus oralis* | 85.0 | 8.7 | *Rothia mucilaginosa* | 97.4 | 6.9 |
| *Veillonella dispar* | 84.8 | 5.5 | *Rothia mucilaginosa* | 82.1 | 2.9 | *Veillonella dispar* | 85.0 | 4.7 | *Granulicatella adiacens* | 94.7 | 3.7 |
| *Streptococcus salivarius* | 84.8 | 4.8 | *Streptococcus parasanguinis* | 74.4 | 5.8 | *Granulicatella adiacens* | 85.0 | 3.1 | *Veillonella dispar* | 92.1 | 7.5 |
| *Streptococcus parasanguinis* | 82.6 | 4.4 | *Prevotella melaninogenica* | 74.4 | 4.5 | ***Pseudomonas aeruginosa*** | 80.0 | 12.0 | *Streptococcus salivarius* | 81.6 | 9.4 |
| *Veillonella parvula* | 82.6 | 2.1 | *Gemella parahaemolysans* | 71.8 | 2.0 | *Rothia mucilaginosa* | 80.0 | 2.1 | *Prevotella melaninogenica* | 81.6 | 5.0 |
| *Rothia dentocariosa* | 82.6 | 1.2 | *Schaalia odontolytica* | 71.8 | 1.7 | *Streptococcus salivarius* | 72.5 | 2.9 | *Schaalia odontolytica* | 76.3 | 1.8 |
| *Prevotella salivae* | 80.4 | 1.2 | *Veillonella parvula* | 61.5 | 1.9 | *Gemella parahaemolysans* | 72.5 | 1.5 | *Gemella parahaemolysans* | 68.4 | 2.1 |
| *Prevotella melaninogenica* | 76.1 | 4.0 | *Rothia dentocariosa* | 61.5 | 1.1 | ***Staphylococcus aureus*** | 67.5 | 12.2 | *Prevotella histicola* | 63.2 | 3.1 |
| ***Staphylococcus aureus*** | 76.1 | 3.8 | *Granulicatella adiacens* | 59.0 | 0.6 | ***Haemophilus influenzae*** | 65.0 | 2.3 | *Campylobacter concisus* | 60.5 | 0.7 |
| *Gemella parahaemolysans* | 76.1 | 1.4 | *Lancefieldella parvula* | 59.0 | 0.3 | *Schaalia odontolytica* | 65.0 | 1.1 | *Stomatobaculum longum* | 60.5 | 0.6 |
| *Granulicatella adiacens* | 73.9 | 0.9 | ***Pseudomonas aeruginosa*** | 56.4 | 17.8 | *Rothia dentocariosa* | 60.0 | 0.8 | *Rothia dentocariosa* | 57.9 | 1.5 |
| *Lancefieldella parvula* | 71.7 | 0.1 | *Prevotella histicola* | 53.8 | 2.2 | *Prevotella melaninogenica* | 57.5 | 4.0 | *Prevotella salivae* | 57.9 | 0.8 |
| *Oribacterium sinus* | 69.6 | 0.2 | ***Haemophilus influenzae*** | 53.8 | 1.5 | *Veillonella parvula* | 55.0 | 1.7 | *Lancefieldella parvula* | 57.9 | 0.7 |
| *Prevotella oris* | 67.4 | 1.2 | *Prevotella pallens* | 53.8 | 0.5 | *Prevotella salivae* | 52.5 | 0.9 | *Veillonella parvula* | 55.3 | 1.2 |
| *Prevotella pallens* | 67.4 | 1.1 | ***Staphylococcus aureus*** | 51.3 | 6.3 |  |  |  | *Oribacterium sinus* | 55.3 | 0.6 |
| ***Haemophilus influenzae*** | 67.4 | 1.0 |  |  |  |  |  |  | *Streptococcus oralis* | 52.6 | 3.4 |
| *Actinomyces naeslundii* | 67.4 | 0.4 |  |  |  |  |  |  | ***Haemophilus influenzae*** | 52.6 | 3.1 |
| *Prevotella histicola* | 65.2 | 3.7 |  |  |  |  |  |  | *Lachnoanaerobaculum orale* | 52.6 | 0.9 |
| *Streptococcus peroris* | 65.2 | 2.7 |  |  |  |  |  |  |  |  |  |
| *Schaalia odontolytica* | 65.2 | 0.7 |  |  |  |  |  |  |  |  |  |
| ***Achromobacter xylosoxidans*** | 63.0 | 1.5 |  |  |  |  |  |  |  |  |  |
| *Scardovia wiggsiae* | 63.0 | 0.2 |  |  |  |  |  |  |  |  |  |
| ***Burkholderia cepacia* complex** | 56.5 | 2.0 |  |  |  |  |  |  |  |  |  |
| *Fusobacterium nucleatum* | 56.5 | 0.6 |  |  |  |  |  |  |  |  |  |
| *Anaeroglobus micronuciformis* | 56.5 | 0.5 |  |  |  |  |  |  |  |  |  |
| *Prevotella denticola* | 56.5 | 0.4 |  |  |  |  |  |  |  |  |  |
| *Campylobacter concisus* | 56.5 | 0.4 |  |  |  |  |  |  |  |  |  |
| *Neisseria cinerea* | 54.3 | 0.9 |  |  |  |  |  |  |  |  |  |
| *Lautropia mirabilis* | 54.3 | 0.4 |  |  |  |  |  |  |  |  |  |
| *Solobacterium moorei* | 54.3 | 0.1 |  |  |  |  |  |  |  |  |  |
| *Actinomyces graevenitzii* | 52.2 | 0.3 |  |  |  |  |  |  |  |  |  |
| *Lachnoanaerobaculum orale* | 52.2 | 0.1 |  |  |  |  |  |  |  |  |  |
