# Supplementary material for: Remodelling of cystic fibrosis respiratory microbiota in response to extended elexacaftor–tezacaftor–ivacaftor therapy
Source: Microbiome. 2026 May 30;14:192. doi: 10.1186/s40168-026-02440-7 (PMC13430856; doi:10.1186/s40168-026-02440-7)
Supplement: Supplementary file 7 — Supplementary Material 6: Table S3 PERMANOVA summary statistics for all groups taken pairwise. Summary statistics are given for Sørensen indices of similarity between groups F-statistic is given in the upper triangle and significance (P) in the lower triangle. Tests with significant differences are highlighted in green. [file 40168_2026_2440_MOESM6_ESM.docx]

**Table S3** PERMANOVA summary statistics for all groups taken pairwise. Summary statistics are given for Sørensen indices of similarity between groups *F*-statistic is given in the upper triangle and significance (*P*) in the lower triangle. Tests with significant differences are highlighted in green.

|  |  | **Pre-ETI** |  |  | **Non-CF** | **On-ETI** |  |  |  |
| --- | --- | --- | --- | --- | --- | --- | --- | --- | --- |
|  |  | **Severe** | **Moderate** | **Mild** | **Healthy** | **6M** | **1Y** | **2Y** | **3Y** |
| **Pre-ETI** | **Severe** |  | 1.51 | 2.90 | 8.25 | 8.11 | 4.39 | 6.15 | 4.95 |
|  | **Moderate** | 0.0877 |  | 1.84 | 7.59 | 6.14 | 3.62 | 6.51 | 5.06 |
|  | **Mild** | 0.0011 | 0.0327 |  | 5.99 | 4.07 | 1.82 | 3.83 | 3.94 |
| **Non-CF** | **Healthy** | <0.0001 | <0.0001 | <0.0001 |  | 6.49 | 5.89 | 6.84 | 8.31 |
| **On-ETI** | **6M** | <0.0001 | <0.0001 | 0.0002 | <0.0001 |  | 2.64 | 6.14 | 6.83 |
|  | **1Y** | <0.0001 | 0.0003 | 0.0249 | <0.0001 | 0.0038 |  | 1.87 | 3.44 |
|  | **2Y** | <0.0001 | <0.0001 | <0.0001 | <0.0001 | <0.0001 | 0.0302 |  | 2.76 |
|  | **3Y** | <0.0001 | <0.0001 | 0.0002 | <0.0001 | <0.0001 | <0.0001 | 0.0018 |  |
