# Supplementary material for: Remodelling of cystic fibrosis respiratory microbiota in response to extended elexacaftor–tezacaftor–ivacaftor therapy
Source: Microbiome. 2026 May 30;14:192. doi: 10.1186/s40168-026-02440-7 (PMC13430856; doi:10.1186/s40168-026-02440-7)
Supplement: Supplementary file 8 — Supplementary Material 7: Table S4 Wilcoxon signed-rank test summary statistics for measures of lung function, diversity and dominance between paired pre- and on-ETI therapy samples when stratified by pre-ETI disease severity status. Summary statistics are given for lung function (%FEV1), Shannon index of diversity, Simpson’s index of diversity, and Berger-Parker index of dominance. Given for each test is the test statistic V, Expected V, variance, and significance (P). Tests with significant differences are highlighted in green. [file 40168_2026_2440_MOESM7_ESM.docx]

**Table S4** Wilcoxon signed-rank test summary statistics for measures of lung function, diversity and dominance between paired pre- and on-ETI therapy samples when stratified by pre-ETI disease severity status. Summary statistics are given for lung function (%FEV_1_), Shannon index of diversity, Simpson’s index of diversity, and Berger-Parker index of dominance. Given for each test is the test statistic *V*, Expected V, variance, and significance (*P*). Tests with significant differences are highlighted in green.

|  | ^Severe^ | | |  | ^Moderate^ | | |  | ^Mild^ | | | |
| --- | --- | --- | --- | --- | --- | --- | --- | --- | --- | --- | --- | --- |
|  | *^V^* | ^Expected V^ | ^Variance^ | *^P^* | *^V^* | ^Expected V^ | ^Variance^ | *^P^* | *^V^* | ^Expected V^ | ^Variance^ | *^P^* |
| ^%FEV^_1_ | ^9.0^ | ^162.5^ | ^1380.0^ | ^< 0.0001^ | ^103.0^ | ^351.5^ | ^4389.3^ | ^0.0002^ | ^1.5^ | ^85.5^ | ^5253.6^ | ^0.0002^ |
| ^Shannon index^ | ^156.0^ | ^162.5^ | ^1381.3^ | ^0.874^ | ^323.0^ | ^370.5^ | ^4754.8^ | ^0.500^ | ^22.0^ | ^95.0^ | ^617.5^ | ^0.002^ |
| ^Simpson's index^ | ^138.0^ | ^162.5^ | ^1381.3^ | ^0.525^ | ^297.0^ | ^370.5^ | ^4754.8^ | ^0.293^ | ^29.0^ | ^95.0^ | ^617.5^ | ^0.006^ |
| ^Berger-Parker index^ | ^179.0^ | ^162.5^ | ^1381.3^ | ^0.672^ | ^572.0^ | ^370.5^ | ^4754.8^ | ^0.003^ | ^120.0^ | ^95.0^ | ^617.5^ | ^0.332^ |
