# Supplementary material for: Remodelling of cystic fibrosis respiratory microbiota in response to extended elexacaftor–tezacaftor–ivacaftor therapy
Source: Microbiome. 2026 May 30;14:192. doi: 10.1186/s40168-026-02440-7 (PMC13430856; doi:10.1186/s40168-026-02440-7)
Supplement: Supplementary file 9 — Supplementary Material 8: Table S5 Core taxa across adults with CF receiving Azithromycin treatment or not when either pre-ETI or on-ETI therapy. Given are core taxa for all pre-ETI and on-ETI therapy samples, pre-ETI therapy not on and on Azithromycin treatment, and on-ETI therapy not on and on Azithromycin treatment. Distribution (Dis) is the percentage number of samples a given core taxon was detected in, and average relative abundance (Abu) across those samples. Given the length of the ribosomal sequences analysed, species identities should be considered putative. In each instance, taxa are ordered by distribution (from most to least). Canonical CF pathogens are highlighted in bold. [file 40168_2026_2440_MOESM8_ESM.docx]

**Table S5** Core taxa across adults with CF receiving Azithromycin treatment or not when either pre-ETI or on-ETI therapy. Given are core taxa for all pre-ETI and on-ETI therapy samples, pre-ETI therapy not on and on Azithromycin treatment, and on-ETI therapy not on and on Azithromycin treatment. Distribution (Dis) is the percentage number of samples a given core taxon was detected in, and average relative abundance (Abu) across those samples. Given the length of the ribosomal sequences analysed, species identities should be considered putative. In each instance, taxa are ordered by distribution (from most to least). Canonical CF pathogens are highlighted in bold.
